# Mutation Rates in Plastid Genomes: They Are Lower than You Might Think

**DOI:** 10.1093/gbe/evv069

**Published:** 2015-04-13

**Authors:** David Roy Smith

**Affiliations:** Department of Biology, University of Western Ontario, London, ON, Canada

**Keywords:** chloroplast genome, mitochondrial DNA, mutation rate, plastid DNA, synonymous substitution

## Abstract

Within plastid-bearing species, the mutation rate of the plastid genome is often assumed to be greater than that of the mitochondrial genome. This assumption is based on early, pioneering studies of land plant molecular evolution, which uncovered higher rates of synonymous substitution in plastid versus mitochondrial DNAs. However, much of the plastid-containing eukaryotic diversity falls outside of land plants, and the patterns of plastid DNA evolution for embryophytes do not necessarily reflect those of other groups. Recent analyses of plastid and mitochondrial substitution rates in diverse lineages have uncovered very different trends than those recorded for land plants. Here, I explore these new data and argue that for many protists the plastid mutation rate is lower than that of the mitochondrion, including groups with primary or secondary plastids as well as nonphotosynthetic algae. These findings have far-reaching implications for how we view plastid genomes and how their sequences are used for evolutionary analyses, and might ultimately reflect a general tendency toward more efficient DNA repair mechanisms in plastids than in mitochondria.

## Introduction

For better or worse, our understanding of plastid biology is largely shaped by studies of land plants. This is particularly evident for plastid genetics. For example, >540 of the 679 complete plastid genome sequences in GenBank, as of January 1, 2015, come from embryophytes, despite the fact that most of the known plastid-containing diversity is represented by protists (Keeling 2010). Nevertheless, major insights into plastid genomes have come from land plants (Wicke et al. 2011), not the least of which is that plastid mutation rates can exceed those of mitochondria.

More than 25 years ago, Wolfe et al. (1987) compared plastid, mitochondrial, and nuclear DNA (ptDNA, mtDNA, and nucDNA) sequences of various land plants and found the silent site substitution rate of the mitochondrion to be lower than those of the plastid and nucleus. When noncoding and synonymous sites (collectively called silent sites) are assumed to be neutrally evolving, the silent site divergence (*d*_silent_) between species or distinct populations can provide an entrée into mutation rate (Kimura et al. 1983). It was, therefore, concluded that the relative levels of *d*_silent_ in land plants reflected a lower mutation rate in the mitochondrion than in the plastid or nucleus (Wolfe et al. 1987). At the time of publication, these findings went against the prevailing notion, based on studies of animal genomes, that mtDNA had a high mutation rate (Brown et al. 1979).

Subsequent investigations (Drouin et al. 2008; Richardson et al. 2013; Zhu et al. 2014) have supported the conclusions of Wolfe et al. (1987), and it is generally accepted that for land plants the plastid and nuclear genomes have an ∼3- to 10-fold greater mutation rate than the mitochondrial genome, with some notable exceptions (Sloan, Alverson, Chuckalovcak, et al. 2012; Zhu et al. 2014). Consequently, it is sometimes assumed that lineages outside land plants have higher rates of mutation in their plastids as compared with their mitochondria. But recent organelle genome analyses from diverse lineages suggest that the opposite is true. To better understand organelle mutational patterns in plastid-bearing eukaryotes, I examined the available substitution rate data for plastid, mitochondrial, and nuclear genomes and found that an mtDNA/ptDNA mutation rate ratio of >1 can be observed in a diversity of eukaryotic lineages and might represent the norm for plastid-bearing species. If true, this could be explained by more efficient DNA repair mechanisms in plastid versus mitochondrial genomes.

### Analyzing Substitution Rates of Eukaryotic Algae

Until recently, little was known about the relative rates of substitution in plastid, mitochondrial, and nuclear genomes of non-land plant species. This is because the data needed for these types of analyses are difficult to generate, requiring nucleotide sequences from three different genetic compartments for at least two distinct “species” or populations. Moreover, the two species must be closely enough related that the silent site divergence, in all three compartments, has not reached saturation (Kimura et al. 1983)—not trivial requirements when considering that most protists are poorly sampled and poorly studied. However, improvements in high-throughput sequencing technologies and a growing interest in microbial eukaryotes, especially those from marine environments (Keeling et al. 2014), mean that organelle and nuclear genomic data are accumulating for a variety of algal groups (Smith 2012, 2013), allowing for accurate measurements of *d*_silent_.

Relative silent site substitution rate statistics are now available for various “primary” algae (i.e., those whose plastids descend directly from the endosymbiosis of a cyanobacterium), including green algae, red algae, and glaucophytes, as well from groups that acquired their plastids through eukaryotic–eukaryotic endosymbioses, such as haptophytes, stramenopiles, and apicomplexans, all of which have red–algal-derived plastids (Keeling 2010) (table 1). In many cases, these data encompass all three genetic compartments, and are based on analyses of whole organelle genomes and at least 25 nuclear genes of diverse function (table 1). It is important to stress, however, that relative substitution rates do not necessarily reflect absolute substitution rates, which to calculate requires knowledge of the number of generations separating the species being compared. But relative rates do provide an estimate of the frequency of silent site substitutions among compartments within a species, and in cases were *d*_silent_ is exceptionally high or low in one compartment, it can be an indication of a high or low absolute rate of substitution.

**Table 1:** Plastid, Mitochondrial, and Nuclear DNA Substitution Rate Statistics from Diverse Plastid-Bearing Lineages

Lineage	Substitutions Per Site												*d*_N_/*d*_S_			*d*_S_ Ratio
	Synonymous			Nonsynonymous			Functional RNA			Intergenic						
	pt	mt	nuc	pt	mt	nuc	pt	mt	nuc	pt	mt	nuc	pt	mt	nuc	pt:mt:nuc
Archaeplastids																
Glaucophytes																
*Cyanophora*	1.01 (1.22)	5.29* (3.17)	1.21 (0.83)	0.03	0.14	0.04	0.007	0.09	–	–	–	–	0.04	0.02	0.06	1:5.3:1.2
Green algae																
*Chlamydomonas*	0.30 (0.11)	0.29 (0.05)	0.37 (0.29)	—	0.01	0.02	—	—	—	0.67	0.38	—	—	0.04	0.06	1:1:1.2
*Dunaliella*	0.09 (0.32)	1.16* (0.52)	—	0.005	0.043	—	0.06	0.11	—	—	—	—	0.09	0.04	—	1:12.9:—
*Mesostigma*	0.11 (0.06)	0.17* (0.11)	0.27 (0.18)	—	—	—	—	—	—	0.03	0.3	—	—	—	—	1:1.5:2.5
*Ostreococcus*	0.76 (0.68)	4.24* (2.48)	1.68 (2.15)	0.03	0.15	0.07	—	—	—	0.81	1.54	—	0.04	0.02	0.05	1:>5:1.9
Land plants																
Angiosperms	0.39 (0.01)	0.13* (0.01)	2.11 (0.09)	0.05	0.02	0.05	—	—	—	—	—	—	—	—	—	1:0.3:5.4
Gymnosperms	0.61 (0.03)	0.28* (0.02)	1.23 (0.09)	0.09	0.07	0.04	—	—	—	—	—	—	—	—	—	1:0.5:2
Red algae																
*Porphyra*	0.47 (0.22)	1.76* (0.58)	0.43 (0.18)	0.03	0.09	0.01	0.01	0.06	0.04	0.17	0.25	0.15	0.06	0.05	0.04	1:3.7:0.9
Haptophytes																
*Emiliania*	0.001 (0.005)	0.01* (0.008)	—	0.0001	0.0004	—	0.0001	0.001	—	0.0008	0.027	—	—	0.09	—	1:10:—
*Phaeocystis*	0.25 (0.16)	2.41* (0.97)	0.85 (0.54)	0.01	0.09	0.05	0.005	0.18	0.006	—	—	—	0.06	0.05	0.07	1:9.6:3.4
Stramenopiles																
*Heterosigma*	0.001 (0.003)	0.012* (0.01)	—	0.0003	0.001	—	0	0.002	—	0.007	0.059	—	0.21	0.11	—	1:12:—
*Nannochloropsis*	0.07 (0.05)	0.08 (0.04)	—	0.004	0.005	—	0.004	0.005	—	—	—	—	0.11	0.07	—	1:1.1:—

Note.—Sources for the data are listed in the Materials and Methods. Abbreviations are as follows: plastid DNA (pt), mitochondrial DNA (mt), and nuclear DNA (nuc); rRNA- and/or tRNA-coding regions (functional RNA); the ratio of nonsynonymous to synonymous substitutions per site (*d*_N_/*d*_S_); the relative synonymous site substitution rate among plastid, mitochondrial, and nuclear DNAs (*d*_S_ ratio); data not available (—). Substitution rates at synonymous, nonsynonymous, and intergenic sites as well as *d*_N_/*d*_S_ are based on averages among loci, except for the intergenic substitutions of *Emiliania*, *Heterosigma*, and *Porphyra*, which are based on concatenated data sets. Substitutions at functional RNA-coding sites are all based on concatenated data sets. Asterisk (*) denotes a significance difference (*P* < 0.05) in the distribution of plastid versus mitochondrial synonymous substitution rates. Bracketed value next to synonymous substitution rate is the standard deviation, expect for angiosperms and gymnosperms where it represents the standard error (Drouin et al. 2008). In some cases, genes showing extreme synonymous site saturation were removed from the analyses (supplementary tables S1–S3, Supplementary Material online).

**Fig. 1.—:**
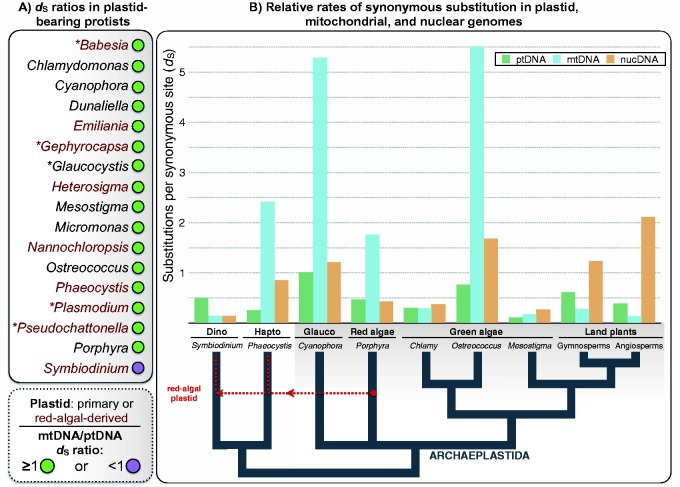
Relative rates in plastid-bearing protists. (*A*) The relative rate of synonymous substitutions in mitochondrial versus plastid genomes (*d*_S_ ratio) for various plastid-bearing protists. An mtDNA/ptDNA *d*_S_ ratio of ≥1 is shown with a green circle and <1 with a purple circle. (*B*) Synonymous substitution rates in plastid, mitochondrial, and nuclear genomes. ptDNA is shown in green, mtDNA in blue, and nucDNA in orange. The Archaeplastida comprises glaucophytes (Glauco), red algae, green algae, and land plants, all of which have primary plastids. The haptophyte (Hapto) *Phaeocystis* and the dinoflagellate (Dino) *Symbiodinium* have secondary, red–algal-derived plastids. The methods used to estimate *d*_S_ and the number and type of loci investigated sometimes differed among the various taxa. The ML approach was used to estimate *d*_S_ for all taxa without an asterisk in front of their names. Alternative methods were used for those with an asterisk (see Materials and Methods).

There exist different methods and models for estimating rates of nucleotide substitution (Li et al. 1985; Yang and Nielsen 2000; Cannarozzi and Schneider 2012). The available substitution rate statistics for plastid-bearing species were not all derived using the same methodologies. Here, I have tried to focus on studies that employed the maximum-likelihood (ML) method (Yang and Nielsen 2000), which is considered to be among the most accurate methods available for measuring substitution rates, particular between distantly related sequences (Muse 1996; Yang and Nielsen 2000). In some cases, I reanalyzed published data sets using the ML method (see Materials and Methods). Consequently, the silent site substitution rates in table 1 and figure 1*B* were calculated using the ML approach, implemented through the program PAML v4 (Yang 2007)*.* In some instances, however, I did not have easy access to the raw data sets that were used to calculate divergence. In such cases, I do not present the per-site substitution rate values, as they were not always calculated using the ML method. Instead, I simply record whether the observed silent site divergence in the mtDNA exceeded that of the ptDNA, making sure that the same methodologies were used for calculating divergence in both compartments (fig. 1*A*).

### Relative Rates of Silent Site Substitution in Diverse Plastid Lineages

The relative levels of *d*_silent_ differ substantially both among and within plastid-bearing lineages, but with the exception of land plants there is a near-universal tendency for plastid genomes to have lower rates of silent site substitution than their mitochondrial counterparts (table 1 and fig. 1). The same pattern is observed for other categories of nucleotide site as well. For instance, substitutions at nonsynonymous positions (*d*_N_) and regions coding for functional RNAs are consistently lower in plastid than in mitochondrial genomes (table 1). The ratio of nonsynonymous to synonymous substitutions (*d*_N_/*d*_S_), which can be used to gauge the intensity and directionality of natural selection, are generally quite low for both organelle genomes (table 1), indicating strong purifying selection on nonsynonymous sites—but see Preston et al. (2014) and Wicke et al. (2014) for exceptions to this trend.

In many instances, the silent site substitution rate differences between mitochondria and plastids are extreme (table 1 and fig. 1). In the haptophyte genus *Phaeocystis*—the members of which can form massive ocean blooms (Arrigo et al. 1999)—*d*_silent_ of the mtDNA is on average 10 times that of the plastid. Isolates of the halophilic green alga *Dunaliella salina* show a 13-fold greater silent site substitution rate in the mitochondrion versus the plastid. And in the coccolithophore *Emiliania huxleyi*, the average *d*_silent_ of the mtDNA is ∼10 times that of the ptDNA, going up to ∼30 times greater when only intergenic sites are considered.

In other cases, the differences between the mitochondrial and plastid compartments are less severe (table 1 and fig. 1). For example, in the red algal genus *Porphyra* and the glaucophyte genus *Cyanophora*, *d*_silent_ of the mtDNA is ∼4–5 times that of the ptDNA. And for the green algal lineages *Mesostigma* and *Chlamydomonas*, the mitochondrial and plastid genomes have similar rates of silent site substitution, although for *Mesostigma* the rate for the mtDNA is slightly higher than that for the ptDNA.

Even certain nonphotosynthetic species display lower rates of substitution in their ptDNAs than their mtDNAs (fig. 1). For instance, a large-scale population genomics study of the malaria parasite *Plasmodium falciparum* (Apicomplexa), involving the sequencing of complete organelle DNAs from 711 distinct isolates, exposed ∼4 times more polymorphisms per silent site in the mtDNA as compared with the ptDNA (Preston et al. 2014). Similarly, organelle genome sequencing of geographical isolates of the apicomplexan parasite *Babesia bovis* uncovered no polymorphisms in the ptDNA, which is ∼35 kb long (∼15 kb of which are silent sites), whereas the mtDNA, which is ∼6 kb long with only ∼3 kb of silent sites, contained multiple polymorphisms (Smith and Keeling 2012).

The substitution rate data from the nuclear compartment are less variable than those from the organelles, but as with the plastid-versus-mitochondrial comparisons, *d*_silent_ for the ptDNA is consistently lower or similar to that of the nucDNA (table 1 and fig. 1). This is true across a wide breadth of plastid-bearing eukaryotes, although for *Porphyra* red algae and the dinoflagellate *Symbiodinium* the ptDNA has a slightly higher silent site substitution rate than the nucDNA—but for the former, it is still much lower than the mtDNA.

Given all of this, it is hard to ignore that for a large range of algae, including those with primary or secondary plastids as well as nonphotosynthetic species, ptDNAs consistently have lower levels of *d*_silent_ than mtDNAs. These trends contrast with those observed for many land plants, where plastids typically harbor higher levels of silent site divergence than mitochondria (table 1 and fig. 1).

### Lower Mutation Rates in Plastid Versus Mitochondrial Genomes

If we accept that the relative silent site divergence for genomes within a cell provides a gateway into the relative mutation rates of those genomes, then the data highlighted above have a clear meaning: Many algae have an mtDNA/ptDNA mutation rate ratio of >1, and such a ratio may be common for plastid-bearing eukaryotes as a whole (fig. 1). This is particularly apparent in lineages with red–algal-derived plastids, including haptophytes, stramenopiles, and apicomplexan parasites, where for some species the mtDNA mutation rate is estimated to be 30 times greater than that of the ptDNA (table 1) (Smith and Keeling 2012).

Land plants appear to be an exception among plastid lineages in that many species are predicted to have higher rates of mutation in their ptDNAs versus mtDNAs (table 1 and fig. 1) (Drouin et al. 2008). That said, a number of seed plants have recently been shown to have unprecedentedly high rates of silent site mtDNA substitution (Mower et al. 2007), including certain *Silene* and *Ajuga* species (Sloan, Alverson, Chuckalovcak, et al. 2012; Zhu et al. 2014). In some of these taxa, elevated mitochondrial rates coincide with inflated ptDNA divergence (Sloan, Alverson, Wu, et al. 2012), but for both *Silene* and *Ajuga*, the mitochondrial rates are higher than those of the plastid (Zhu et al. 2014). Even for land plant mtDNAs with very low levels of *d*_silent_, the substitution rate measurements are often based entirely on synonymous sites (Drouin et al. 2008), and the divergence at noncoding sites can be much higher but often goes unrecorded because these regions can be difficult to align (Christensen 2013). This suggests that for land plants an mtDNA/ptDNA mutation rate ratio of >1 might not be as uncommon as once thought.

Why do plastid genomes consistently exhibit lower rates of silent site substitution and have lower predicted mutation rates than their mitochondrial neighbors? Or, rather, why do mitochondria typically have higher mutation rates than plastids? In other respects, mitochondrial and plastid DNAs are very similar. Both are usually uniparentaly inherited and housed in energy-producing organelles, which evolved from the endosymbiosis of free-living bacteria more than a billion years ago; both are highly reduced and have lost or transferred most of their genes to the host nuclear genome; and both are dependent on nuclear-encoded, organelle-targeted proteins for the preservation of crucial biochemical pathways and for essential repair-, replication-, and expression-related functions. Moreover, in plants and algae, various DNA repair, replication, and recombination proteins targeted to the mitochondrion have plastid-targeted paralogs (Sloan and Taylor 2012), and a significant proportion are targeted to both organelles (Carrie et al. 2009).

The nature and accuracy of these organelle-targeted DNA maintenance machineries can have a huge impact on organelle mutation rates (Sloan and Taylor 2012). The fact that parts of these machineries are shared between mitochondria and plastids can explain why some species exhibit similar mtDNA and ptDNA mutation rates. But when considering that the origin and evolution of organelle DNA repair and recombination processes involves a complex history of gene transfer, co-option, duplication, and replacement events, it is not surprising that organelle mutation rates can vary both within and between species (Sloan and Taylor 2012; Barnard-Kubow et al. 2014). Indeed, mitochondria boast some of the highest (Oliveira et al. 2008) and lowest (Richardson et al. 2013) mutation rate estimates of any eukaryotic or bacterial genome.

One explanation for the substitution rate patterns described here is that for a number of eukaryotes the fidelity and efficacy of mitochondrial maintenance machineries are more variable and capricious than those of plastids (Lynch et al. 2006; Richardson et al. 2013; Smith, Jackson, et al. 2014; Zhu et al. 2014). Support for this hypothesis not only comes from the observation that substitution rates are often higher in mitochondrial versus plastid genomes, but also from the observation that mitochondrial chromosomes display a greater breadth of complexity and more severe genomic embellishments than those of plastids (Smith and Keeling 2015). It is thought that the propensity for mtDNAs to adopt such “extreme” architectures has its roots in wayward DNA maintenance pathways (Davila et al. 2011; Christensen 2013; Smith and Keeling 2015).

Another difference between mitochondria and plastids, which could help explain why the former are prone to higher substitution rates, is that mtDNAs generally contain fewer genes than ptDNAs. Although speculative, a smaller gene complement could permit larger fluctuations in mutation rate because selection against a mutator allele would be proportional to its effect on the rate of deleterious mutations per genome, not per nucleotide (Smith and Keeling 2015).

### Implications of a Low Plastid/Mitochondrial Mutation Rate

There are over 5,000 mitochondrial and plastid genome sequences in the National Center for Biotechnology Information database, making organelle genomes among the most highly sequenced chromosomes. From unraveling the population genetics of malaria parasites (Preston et al. 2014) to developing biofuels (Hannon et al. 2010) to tracking the history of ancient Arctic vegetation (Willerslev et al. 2014), mtDNAs and ptDNAs are among the most important and widely used genetic markers. They have shaped our understanding of eukaryotic evolution (Keeling 2010; Williams et al. 2013), and been pivotal in the fields of archaeology (Orlando 2014), forensics (Budowle et al. 2003), and medicine (Dahl and Rosenthal 2008). It is therefore paramount that we understand the mutational processes impacting these genomes.

Land plant studies have improved our knowledge of organelle genetics, but the patterns of organelle genome evolution in plants do not necessarily reflect those of other lineages. If it is true that for many microbial eukaryotes the plastid mutation rate is lower than that of the mitochondrion, it could mean that ptDNAs are a more suitable genome for wide-scale comparative analyses, such as those attempting to resolve relationships among distantly related groups or organisms (Baurain et al. 2010). Conversely, mitochondrial genomes, with their proclivity toward higher mutation rates, could be useful for fine-scale genetic analyses, such as population genetic studies (Preston et al. 2014).

Again, it is important to stress that the studies from which the mtDNA and ptDNA substitution rate data derive sometimes differed in the type and number of loci used and in the methodologies employed for calculating divergence. These differences as well as the differences in organelle genomic architecture within and among the species being compared should be taken into consideration when assessing the major trends presented in this study. As more data on relative rates emerge from poorly studied plastid-containing lineages, they will likely provide an even more dynamic picture of organelle and nuclear mutation rates. It will be interesting to see if algal lineages not included in this study, such as euglenophytes and chlorarachniophytes, both of which have green–algal-derived plastids, also have lower rates of silent site substitution in their plastid versus mitochondrial compartments. I predict that they will.

## Materials and Methods

Relative substitution rate data were derived or taken directly from the following sources and species/strain comparisons: *B. bovis* C9.1 versus *B. bovis* T2Bo (Smith and Keeling 2012); *Chlamydomonas reinhardtii* versus *Chlamydomonas globosa* SAG 7.73 (formerly called *Chlamydomonas incerta* SAG 7.73) (Popescu and Lee 2007); *Cyanophora paradoxa* strain NIES-763 versus CCMP329 (Smith, Jackson, et al. 2014); *D. salina* CCAP 19/18 versus *D. salina* CONC-001 (Del Vasto et al. 2015); *E. huxleyi* CCMP373 versus *E. huxleyi* CCMP1516 (Smith and Keeling 2012); *Gephyrocapsa oceanica* (dozens of geographical isolates) (Bendif et al. 2014); *Glaucocystis* spp. (∼10 geographical isolates) (Chong et al. 2014); *Heterosigma akashiwo* CCMP452 versus *H. akashiwo* NIES-293 (Cattolico et al. 2008; Smith and Keeling 2012); land plants (27 different species) (Drouin et al. 2008); *Mesostigma viride* strain NIES-296 versus SAG 50-1 (Hua et al. 2012); *Micromonas pusilla* CCMP1545 versus *Micromonas* sp. RCC299 (Worden et al. 2009) (supplementary table S1, Supplementary Material online); *Nannochloropsis gaditana* CCMP527 versus *Nannochloropsis salina* CCMP1776 (Starkenburg et al. 2014) (supplementary table S2, Supplementary Material online); *Ostreococcus* sp. RCC809 versus *Ostreococcus tauri* OTTH0595 (supplementary table S3, Supplementary Material online); *Phaeocystis antarctica* CCMP1374 versus *Phaeocystis globosa* Pg-G(A) (Smith, Arrigo, et al. 2014); *P. falciparum* (hundreds of geographical isolates) (Preston et al. 2014); *Porphyra umbilicalis* UTEX LB 2951 versus *Porphyra purpurea* strain “Avonport” (Smith et al. 2012); *Pseudochattonella* sp. JG8 versus *Pseudochattonella farcimen* UIO109 versus *Pseudochattonella verruculosa* NIES-670 (Riisberg and Edvardsen 2014); and *Symbiodinium* sp. A2 versus C90 (Pochon et al. 2014; Smith and Keeling 2015). Note: The data sets for *Emiliania*, *Heterosigma*, and *Symbiodinium* (Smith and Keeling 2012; Pochon et al. 2014) were reanalyzed using the ML method, as described below.

In cases where the substitution rate statistics were recalculated or did not come directly from the literature (e.g., *Micromonas*, *Nannochloropsis*, and *Ostreococcus*; supplementary tables S1–S3, Supplementary Material online), they were estimated as follows. Organelle and nuclear genes were aligned with MUSCLE (Edgar 2004), implemented through Geneious v7.1.4 (Biomatters Ltd, Auckland, NZ), using default settings. Synonymous and nonsynonymous substitutions were measured with the CODEML program of PAML v4.3 (Yang 2007), employing the ML method, the F3 × 4 codon model of Goldman and Yang (1994) (options: seqtype = 1, runmode = −2, and CodonFreq = 2 in the codeml.ctl file), and making the proper adjustments for changes in the genetic code. Substitutions in non–protein-coding regions were estimated with BASEML of PAML, using the HKY85 model. Significance levels (*P* values) of mtDNA versus ptDNA substitution rate differences were taken from the primary literature or calculated using the *t*-test as implemented in Microsoft Excel (Mac) 2011 v14.4.4.

## Supplementary Material

Supplementary tables S1–S3 are available at *Genome Biology and Evolution* online (http://www.gbe.oxfordjournals.org/).

Supplementary Data
