# Supplementary material for: Mutation Rates in Plastid Genomes: They Are Lower than You Might Think
Source: Genome Biol Evol. 2015 Apr 13;7(5):1227–34. doi: 10.1093/gbe/evv069 (PMC4453064; doi:10.1093/gbe/evv069)
Supplement: Supplementary Data [file supp_7_5_1227__index.html]

Mutation rates in plastid genomes: they are lower than you might think. — Mutation Rates in Plastid Genomes: They Are Lower than You Might Think — Supplementary Data 

# Mutation Rates in Plastid Genomes: They Are Lower than You Might Think

## Supplementary Data

files

**Files in this Data Supplement:**

- Supplementary Data - pdf file
