# Supplementary material for: Mutation Rates in Plastid Genomes: They Are Lower than You Might Think
Source: Genome Biol Evol. 2015 Apr 13;7(5):1227–34. doi: 10.1093/gbe/evv069 (PMC4453064; doi:10.1093/gbe/evv069)
Supplement: Supplementary Data [file supp_evv069_Supplementary_Data_R1.pdf]

Supplementary Materials for:

Mutation rates in plastid genomes: they are lower than you might think.

David Roy Smith[1,*]

[1]Department of Biology, University of Western Ontario, London, ON, N6A 5B7, Canada.

*Corresponding author: David Roy Smith, Department of Biology, University of Western Ontario, London, Ontario, N6A 5B7, Canada. Email: dsmit242@uwo.ca. Tel: (519) 661 2111, ext.86482.

Table of Contents

Supplementary Table S1. Nucleotide substitution rates between *Micromonas pusilla* CCMP1545 and *Micromonas* sp. RCC299. .... **2**
- **Part A. Mitochondrial DNA-located genes.** **2**
- **Part B. Plastid DNA-located genes** **2**

Supplementary Table S2. Nucleotide substitution rates between *Nannochloropsis gaditana* CCMP527 and *N. salina* CCMP1776. .... **3**
- **Part A. Mitochondrial DNA-located genes.** **3**
- **Part B. Plastid DNA-located genes** **4**

Supplementary Table S3. Nucleotide substitution rates between *Ostreococcus* sp. RCC809 and *Ostreococcus tauri* OTTH0595. .... **7**
- **Part A. Mitochondrial DNA-located genes.** **7**
- **Part B. Plastid DNA-located genes** **8**
- **Part C. Nuclear DNA-located genes** **10**
- **Part D. Mitochondrial DNA intergenic regions.** **12**
- **Part E. Plastid DNA intergenic regions.** **12**

Supplementary Table S1. Nucleotide substitution rates between *Micromonas pusilla* CCMP1545 and *Micromonas* sp. RCC299.

Part A. Mitochondrial DNA-located genes.

**MtDNA gene**	**# codons**	d_N / d_S	d_N	d_S
atp6	249	0.012	0.155	13.366
atp9	73	0.001	0.012	12.069
cob	385	0.008	0.073	9.608
cox1	527	0.013	0.099	7.580
cox2	262	0.013	0.126	9.970
cox3	265	0.002	0.122	61.852
nad1	332	0.001	0.072	60.342
nad2	495	0.029	0.365	12.791
nad3	116	0.002	0.112	50.550
nad4	485	0.002	0.141	60.250
nad4L	99	0.049	0.125	2.561
nad5	670	0.021	0.201	9.465
nad6	262	0.032	0.383	11.802
nad7	397	0.016	0.088	5.595
nad9	177	0.003	0.212	69.797
rpl14	123	0.034	0.284	8.347
rps12	122	0.045	0.148	3.318
rps14	98	0.010	0.448	43.854
rps19	83	0.006	0.351	62.796
**Average**	**274.74**	**0.02**	**0.19**	**27.15**
**Standard deviation**	**177.13**	**0.02**	**0.12**	**25.24**

Part B. Plastid DNA-located genes.

**PtDNA genes**	**# codons**	d_N / d_S	d_N	d_S
psaA	750	0.006	0.002	0.312
psaB	733	0.009	0.004	0.383
psbA	352	0.053	0.003	0.047
psbE	83	0.024	0.006	0.239
psbF	37	---	0.000	0.000
psbL	34	---	0.026	0.000
psbZ	61	0.157	0.037	0.238
RNA polymerase alpha	351	0.039	0.091	2.329
RNA polymerase beta chain	1184	0.004	0.349	82.106
RNA polymerase beta subunit	1075	0.041	0.097	2.360
RNA polymerase beta' subunit	764	0.059	0.138	2.363

PtDNA genes	# codons	d_N / d_S	d_N	d_S
rpl14	118	0.025	0.015	0.590
rpl16	138	0.053	0.026	0.489
rpl2	276	0.028	0.019	0.664
rpl23	86	0.054	0.011	0.201
rpl5	183	0.076	0.068	0.899
rps11	129	0.018	0.014	0.768
rps12	123	0.011	0.007	0.654
rps18	69	0.046	0.021	0.462
rps19	91	0.018	0.010	0.530
rps3	210	0.050	0.028	0.549
rps7	155	0.017	0.017	0.971
rps8	121	0.028	0.034	1.215
rps9	127	0.041	0.035	0.846
translation initiation factor 1	83	0.019	0.037	1.925
tufA	407	0.028	0.026	0.960
ycf3	165	0.005	0.008	1.421
**Average**	**292.78**	**0.04**	**0.04**	**3.83**
**Standard deviation**	**321.07**	**0.03**	**0.07**	**15.66**

Supplementary Table S2. Nucleotide substitution rates between *Nannochloropsis gaditana* CCMP527 and *N. salina* CCMP1776.

Part A. Mitochondrial DNA-located genes.

MtDNA genes	# codons	d_N / d_S	d_N	d_S
atp1	519	0.0109	0.0008	0.0733
atp6	261	0.0434	0.0017	0.0403
atp8	104	0.0949	0.0084	0.088
atp9	74	0.001	0.0001	0.0779
cob	385	0.0331	0.0011	0.0344
cox1	505	0.054	0.0068	0.1265
cox2	301	0.001	0	0.0283
cox3	269	0.001	0	0.0307
nad1	325	0.0698	0.0055	0.078
nad2	492	0.0367	0.0035	0.0956
nad3	121	0.001	0.0001	0.0512
nad4	524	0.0312	0.0042	0.133
nad4L	97	0.001	0.0001	0.1422
nad5	673	0.0504	0.0036	0.0717
nad6	194	0.0484	0.0046	0.0955
nad7	397	0.0284	0.0033	0.1145
nad9	188	0.001	0.0001	0.0903

MtDNA genes	# codons	d_N / d_S	d_N	d_S
nad11	505	0.1012	0.0101	0.0995
rpl2	274	0.0447	0.0046	0.102
rpl5	178	0.2947	0	0
rpl6	182	0.3321	0.0308	0.0926
rpl14	125	0.001	0	0.013
rpl16	142	0.0548	0.003	0.0541
rps2	211	0.1405	0.0152	0.1084
rps3	419	0.1441	0.0144	0.0998
rps4	240	0.1335	0.0108	0.0809
rps7	144	0.0391	0.0056	0.1436
rps8	127	0.001	0	0.0313
rps10	111	0.2484	0.0238	0.0959
rps11	155	0.0288	0.0058	0.2009
rps12	124	0.001	0.0001	0.0957
rps13	117	0.001	0.0001	0.1202
rps14	97	0.1729	0.0088	0.0506
rps19	84	0.001	0	0.0174
tatC	238	0.0918	0.008	0.0873
**Average**	**254.34**	**0.067**	**0.005**	**0.082**
**Standard deviation**	**159.91**	**0.085**	**0.007**	**0.043**
**Concatenated rRNA genes**	3742 nt		$d_i = 0.005$	

Part B. Plastid DNA-located genes.

PtDNA genes	# codons	d_N / d_S	d_N	d_S
atpA	504	0.016	0.001	0.052
atpB	489	0.011	0.001	0.074
atpD	231	0.078	0.006	0.070
atpE	138	0.194	0.003	0.015
atpF	154	0.183	0.006	0.032
atpG	159	0.114	0.003	0.026
atpH	82	0.000	0.000	0.000
atpI	248	0.043	0.002	0.042
cbbX	335	0.030	0.004	0.124
ccs1	422	0.077	0.006	0.077
ccsA	302	0.074	0.004	0.059
chlB	506	0.038	0.004	0.104
chlI	410	0.077	0.007	0.093
chlL	287	0.019	0.001	0.074
chlN	443	0.038	0.005	0.125
clpA	148	0.386	0.017	0.044
clpC1	383	0.019	0.002	0.115

PtDNA genes	# codons	d_N / d_S	d_N	d_S
clpC2	448	0.061	0.007	0.106
dnaK	601	0.032	0.003	0.085
ftsH	696	0.049	0.003	0.060
groEL	534	0.045	0.007	0.153
ilvB	591	0.083	0.006	0.066
petA	318	0.016	0.001	0.081
petB	214	0.001	0.000	0.029
petD	159	0.001	0.000	0.023
petF	101	0.001	0.000	0.084
petG	33	0.001	0.000	0.040
petJ	120	0.211	0.004	0.016
petL	30	---	0.017	0.000
petM	30	0.001	0.000	0.308
petN	28	0.001	0.000	0.000
psaA	744	0.001	0.000	0.054
psaB	736	0.019	0.001	0.060
psaC	80	0.106	0.005	0.049
psaD	135	0.001	0.000	0.049
psaE	66	3.980	0.000	0.000
psaF	183	0.014	0.002	0.160
psaI	44	0.001	0.000	0.143
psaJ	40	0.001	0.000	0.057
psaL	171	0.035	0.003	0.073
psaM	29	0.000	0.000	0.000
psb28	112	0.124	0.013	0.108
psbA	359	0.001	0.000	0.006
psbB	508	0.001	0.000	0.082
psbC	468	0.017	0.001	0.055
psbD	355	0.001	0.000	0.044
psbE	82	0.270	0.000	0.000
psbF	42	0.001	0.000	0.108
psbH	66	0.001	0.000	0.048
psbI	37	0.404	0.000	0.000
psbJ	38	0.001	0.000	0.043
psbK	43	0.001	0.000	0.027
psbL	41	0.001	0.000	0.000
psbN	51	0.001	0.000	0.023
psbT	31	0.000	0.014	0.000
psbV	161	0.059	0.003	0.042
psbX	38	0.020	0.000	0.000
psbY	35	0.000	0.000	0.000
psbZ	60	0.001	0.000	0.082

**PtDNA genes**	**# codons**	d_N / d_S	d_N	d_S
rbcL	486	0.021	0.001	0.044
rbcS	88	0.169	0.011	0.063
rpl1	224	0.107	0.008	0.070
rpl2	274	0.062	0.005	0.073
rpl3	203	0.001	0.000	0.117
rpl4	253	0.196	0.010	0.052
rpl5	183	0.115	0.002	0.020
rpl6	179	0.045	0.005	0.104
rpl11	141	0.067	0.003	0.044
rpl12	129	0.063	0.003	0.052
rpl13	144	0.073	0.006	0.078
rpl14	120	0.001	0.000	0.130
rpl16	137	0.001	0.000	0.047
rpl18	103	0.419	0.008	0.019
rpl19	121	0.340	0.015	0.043
rpl20	133	0.141	0.006	0.044
rpl21	148	0.191	0.005	0.029
rpl22	153	0.001	0.000	0.104
rpl23	118	0.044	0.004	0.081
rpl27	107	0.123	0.012	0.096
rpl29	70	0.001	0.000	0.034
rpl31	73	0.001	0.000	0.050
rpl32	54	0.048	0.016	0.330
rpl33	63	0.001	0.000	0.061
rpl34	48	0.000	0.008	0.000
rpl35	63	0.001	0.000	0.076
rpl36	36	0.001	0.000	0.133
rpoA	443	0.146	0.008	0.057
rpoB	1054	0.004	0.000	0.095
rpoC1	749	0.066	0.006	0.094
rpoC2	1317	0.069	0.006	0.084
rps2	249	0.045	0.002	0.036
rps3	85	0.072	0.005	0.069
rps4	207	0.026	0.002	0.079
rps5	176	0.076	0.005	0.062
rps6	105	0.001	0.000	0.104
rps7	157	0.049	0.003	0.053
rps8	131	0.112	0.003	0.029
rps9	135	0.001	0.000	0.227
rps10	114	0.393	0.011	0.029
rps11	126	0.001	0.000	0.089
rps12	124	0.001	0.000	0.084

PtDNA genes	# codons	d_N / d_S	d_N	d_S
rps13	122	0.037	0.003	0.086
rps14	99	0.224	0.009	0.041
rps16	83	0.001	0.000	0.030
rps17	84	0.001	0.000	0.072
rps18	71	0.001	0.000	0.021
rps19	94	0.042	0.004	0.101
rps20	95	0.053	0.004	0.083
secA	867	0.041	0.003	0.080
secY	378	0.227	0.007	0.031
tatC	251	0.068	0.005	0.080
thiG	268	0.092	0.005	0.051
thiS	75	0.162	0.016	0.097
tufA	408	0.012	0.001	0.086
ycf3	166	0.001	0.000	0.071
ycf4	194	0.001	0.000	0.142
ycf12	33	0.129	0.000	0.000
ycf16	282	0.131	0.011	0.085
ycf24	496	0.040	0.003	0.085
ycf34	84	0.056	0.015	0.263
ycf36	165	0.117	0.011	0.092
ycf46	498	0.115	0.009	0.082
ycf49	96	0.362	0.014	0.039
ycf54	106	0.927	0.013	0.014
ycf59	346	0.071	0.005	0.069
ycf66	100	0.001	0.000	0.060
**Average**	**225.26**	**0.105**	**0.004**	**0.068**
**Standard deviation**	**221.47**	**0.370**	**0.004**	**0.054**
**Concatenated rRNA genes**	4552 nt		$d_i = 0.004$	

Supplementary Table S3. Nucleotide substitution rates between *Ostreococcus* sp. RCC809 and *Ostreococcus tauri* OTTH0595.

Part A. Mitochondrial DNA-located genes.

MtDNA genes	# codons	d_N / d_S	d_N	d_S
atp1	506	0.016	0.035	2.214
atp6	381	0.009	0.026	2.830
atp8	165	0.005	0.370	79.057
atp9	73	0.001	0.001	0.570
cob	248	0.007	0.024	3.421
cox1	528	0.008	0.019	2.392
cox2	256	0.017	0.050	3.003

MtDNA genes	# codons	d_N / d_S	d_N	d_S
cox3	263	0.032	0.048	1.520
nad1	327	0.007	0.026	3.820
nad2	496	0.022	0.162	7.315
nad3	114	0.004	0.034	8.168
nad4	488	0.015	0.060	4.089
nad4L	99	0.003	0.110	35.642
nad5	667	0.017	0.064	3.819
nad6	255	0.049	0.176	3.604
nad7	393	0.003	0.015	4.937
nad9	184	0.034	0.149	4.415
ndhk	165	0.006	0.035	5.485
rpl14	123	0.023	0.113	4.929
rpl16	132	0.048	0.105	2.208
rpl5	177	0.090	0.320	3.566
rpl6	186	0.003	0.280	85.703
rps10	113	0.005	0.386	74.577
rps11	120	0.041	0.142	3.444
rps12	120	0.022	0.067	3.014
rps13	115	0.030	0.150	5.072
rps14	98	0.003	0.212	79.618
rps19	79	0.002	0.156	77.068
rps2	197	0.043	0.265	6.111
rps3	276	0.012	0.298	25.422
rps4	204	0.021	0.278	13.211
rps7	163	0.064	0.260	4.070
rps8	123	0.072	0.225	3.123
ymf39	179	0.011	0.272	24.845
**Average**	**235.68**	**0.02**	**0.15**	**17.42**[a]
**Standard deviation**	**151.66**	**0.02**	**0.11**	**27.12**

[a]When the eight genes with extremely high d_S values (>24) are removed from the analysis, the average synonymous-site substitution rate drops to 4.24 (standard deviation = 2.48).

Part B. Plastid DNA-located genes.

PtDNA genes	# codons	d_N / d_S	d_N	d_S
atpA	502	0.052	0.026	0.495
atpB	480	0.021	0.014	0.647
atpE	141	0.034	0.044	1.278
atpF	167	0.067	0.046	0.683
atpH	100	0.037	0.004	0.114
atpI	235	0.011	0.006	0.536
clpP	199	0.002	0.004	1.763
infA	77	0.047	0.040	0.854

PtDNA genes	# codons	d_N / d_S	d_N	d_S
petA	306	0.047	0.052	1.110
petB	214	0.019	0.010	0.531
petG	36	0.001	0.000	0.242
psaA	750	0.012	0.004	0.360
psaB	732	0.017	0.009	0.553
psaC	80	0.001	0.001	0.851
psaI	35	0.001	0.001	0.703
psaJ	41	0.069	0.045	0.656
psaM	30	0.001	0.000	0.298
psbA	352	0.037	0.004	0.103
psbB	442	0.018	0.007	0.400
psbC	460	0.037	0.015	0.396
psbD	351	0.011	0.004	0.351
psbE	81	0.115	0.032	0.277
psbF	37	0.001	0.000	0.077
psbH	77	0.033	0.012	0.343
psbI	37	0.001	0.000	0.154
psbJ	40	0.211	0.023	0.110
psbK	44	0.063	0.021	0.330
psbL	37	0.001	0.000	0.110
psbN	43	0.015	0.009	0.611
psbT	30	0.193	0.016	0.082
rbcL	474	0.090	0.021	0.232
rpl14	32	0.162	0.044	0.272
rpl16	137	0.028	0.022	0.799
rpl2	276	0.035	0.028	0.813
rpl20	110	0.037	0.052	1.410
rpl23	86	0.022	0.006	0.253
rpl32	71	0.086	0.097	1.127
rpl36	36	0.001	0.001	1.447
rpl5	183	0.061	0.039	0.637
rpoA	355	0.041	0.122	2.961
rpoB	1087	0.024	0.054	2.293
rpoC1	736	0.055	0.102	1.855
rpoC2	1007	0.066	0.227	3.425
rps11	129	0.005	0.003	0.679
rps12	123	0.009	0.007	0.776
rps14	99	0.033	0.027	0.808
rps19	91	0.017	0.010	0.562
rps2	223	0.071	0.076	1.060
rps3	210	0.036	0.024	0.674
rps4	196	0.049	0.048	0.980

PtDNA genes	# codons	d_N / d_S	d_N	d_S
rps7	155	0.021	0.020	0.962
rps8	121	0.068	0.032	0.472
rps9	126	0.064	0.025	0.391
tufA	408	0.051	0.040	0.785
ycf12	118	0.031	0.011	0.361
ycf3	165	0.001	0.002	2.098
ycf9	61	0.024	0.008	0.307
**Average**	**227.56**	**0.04**	**0.03**	**0.76**
**Standard deviation**	**243.00**	**0.04**	**0.04**	**0.68**

Part C. Nuclear DNA-located genes.

NucDNA genes	# codons	d_N / d_S	d_N	d_S
RNA homing endonuclease	72	0.001	0.000	0.000
Senescence-associated protein	144	0.474	0.000	0.000
Eif4 eukaryotic translation initiation factor	586	0.025	0.343	13.833
psaL photosystem I subunit XI	200	0.019	0.178	9.192
ATP synthase beta chain, mitochondrial precursor	527	0.002	0.096	61.767
ATP synthase delta subunit 2	176	0.048	0.189	3.934
ATP synthase F1 alpha subunit	562	0.039	0.136	3.509
Chloroplast light-harvesting complex I protein precursor Lhca4	235	0.072	0.167	2.310
Chlorophyll a/b-binding protein CP29	234	0.118	0.179	1.521
Dynein 8 kDa light chain flagellar outer arm	90	0.047	0.156	3.315
rpl9	204	0.062	0.154	2.487
ATP synthase mt F1 assembly factor 2	240	0.036	0.122	3.387
histone H3	119	0.025	0.046	1.878
cytochrome P450	522	0.025	0.066	2.647
ubiquinot cytochrome C reductase	57	0.001	0.077	76.563
rpl9	191	0.034	0.116	3.397
rpl28	146	0.060	0.104	1.738
gpsA glycerol-3-phosphate dehydrogenase	172	0.019	0.071	3.678
psbB oxygen-evolving complex-related	352	0.031	0.061	1.975
acetyl-CoA carboxylase	1130	0.036	0.069	1.903
glucose-6-phosphate phosphate translocator 2	300	0.022	0.037	1.691
glutamine amidotransferase cyclase	537	0.033	0.060	1.815
indole-3-glycerol phosphate lysase	271	0.091	0.113	1.241
ubiquinol-cytochrome-c reductase cytochrome c1	272	0.048	0.098	2.032
rpl2	265	0.059	0.064	1.095
Tpt phosphate/phosphoenolpyruvate	302	0.042	0.070	1.639

NucDNA genes	# codons	d_N / d_S	d_N	d_S
translocator				
rpl17	182	0.091	0.095	1.050
chloroplast ribose-5-phosphate isomerase	249	0.019	0.045	2.390
ribulose-1,5-bisphosphate carboxylase small subunit	167	0.107	0.115	1.073
uridine monophosphate synthetase	474	0.029	0.046	1.556
rps1	249	0.058	0.060	1.033
translation elongation factor mitochondrial	420	0.063	0.059	0.932
psaF	228	0.099	0.070	0.707
GTP-binding regulatory protein beta chain	318	0.049	0.049	0.995
rpl27	134	0.033	0.041	1.244
tauri beta tubulin	432	0.012	0.013	1.039
rpl15-2	188	0.033	0.027	0.831
RuBisCO activase	405	0.048	0.039	0.826
rpl3	91	0.066	0.040	0.601
rpl37	128	0.038	0.029	0.755
rps11	299	0.036	0.028	0.770
Ferredoxin-NADP oxidoreductase chloroplast precursor	378	0.067	0.039	0.588
rps29	55	0.045	0.036	0.815
cytochrome b6 f complex subunit4	158	0.026	0.021	0.811
GTP-binding protein sar1	192	0.016	0.013	0.800
rpl35	104	0.043	0.032	0.739
histone H2A	120	0.016	0.011	0.656
rps14	138	0.018	0.010	0.519
adenosylhomocysteinase	481	0.034	0.022	0.641
psaE photosystem I reaction center subunit	53	0.026	0.016	0.601
RNA polymerase II	71	0.039	0.023	0.589
psaD, PSI-D, subunit II, photosystem I protein	131	0.046	0.026	0.566
rpl22	111	0.033	0.029	0.861
rps15	129	0.015	0.010	0.624
EF1 elongation factor 1-alpha	466	0.057	0.028	0.491
H3 and H4	134	0.046	0.020	0.437
rps24	130	0.044	0.022	0.500
rpsL15/L27	146	0.052	0.023	0.447
histone H2B	110	0.005	0.004	0.734
histone H3.3	135	0.001	0.000	0.212
**Average**	**251.87**	**0.05**	**0.07**	**3.93**[a]
**Standard deviation**	**184.50**	**0.06**	**0.06**	**12.47**

[a]When the two genes with extremely high d_S values (>25) are removed from the analysis, the average synonymous-site substitution rate drops to 1.68 (standard deviation = 2.15).

Part D. Mitochondrial DNA intergenic regions.

MtDNA intergenic region	Alignment length (nt)	d_i
cob / trnQ	52	9
trnH / ndhK	507	2.316
atp9 / trnL	60	1.316
atp6 / trnG	36	1.179
nad9 / atp1	55	2.212
nad4 / nad2	54	0.733
nad5 / nad4	48	0.970
nad6 / atp6	63	0.632
ymf39 / cox1	175	0.466
rps11 / atp9	78	0.557
nad2 / cox3	37	0.503
rrs / trnM	38	0.549
trnG / trnR	289	0.423
rps12 / cob	53	0.652
**Average**	**110.36**	**1.54**
**Standard deviation**	**133.65**	**2.23**

Part E. Plastid DNA intergenic regions.

PtDNA intergenic regions	Alignment length (nt)	d_i
psbA-rrl	442	4.448
psaJ-trnF	117	2.275
trnN-ORF1260	194	4.052
petB-trnK	109	1.601
rbcL-petG	149	1.388
atpE-clpP	110	2.023
rpl32-ORF537	164	1.700
trnL-trnE	92	1.224
trnN-trnT	117	0.894
psaI-ycf12	90	0.933
atpA-rps14	195	1.239
psbN-rps4	152	0.828
psbA-petG	232	0.655
ORF1260-psbE	163	0.682
psaB-petA	338	0.616
trnD-psaA	354	0.627
rps2-rrs	119	0.621
psaC-atpB	180	0.954
rpl20-trnS	77	0.618
rps7-tufA	106	0.627

**PtDNA intergenic regions**	**Alignment length (nt)**	d_i
psbT-psbD	204	0.576
clpP-rpl32	209	0.642
atpB-atpE	59	0.650
psbE-psbF	95	0.586
rps19-rps3	73	0.538
trnM-rpl23	202	0.508
atpI-atpH	179	0.476
psbC-trnR	127	0.469
ycf9-trnM	195	0.712
psbI-psaJ	85	0.429
trnC-rpoB	109	0.402
rps9-trnL	119	0.457
rps4-ycf3	158	0.370
trnS-rpl20	171	0.389
trnF-rbcL	125	0.363
psbJ-petB	143	0.344
rps18-trnE	188	0.348
trnK-psaC	99	0.291
trnS-psbK	105	0.291
atpH-atpF	175	0.284
trnM-trnG	367	0.244
tufa-trnC	327	0.224
trnL-atpI	111	0.272
rps14-trnQ	70	0.257
psaM-trnP	137	0.202
atpF-atpA	169	0.192
psbH-psbN	82	0.142
trnV-psbB	280	0.061
**Average**	**163.81**	**0.81**
**Standard deviation**	**84.87**	**0.87**
